# Exploring the Relationship between Biological Maturation Level, Muscle Strength, and Muscle Power in Adolescents

**DOI:** 10.3390/biology11121722

**Published:** 2022-11-28

**Authors:** Hakan Yapici, Mehmet Gulu, Fatma Hilal Yagin, Ozgur Eken, Tomasz Gabrys, Vera Knappova

**Affiliations:** 1 Department of Coaching Education, Faculty of Sport Sciences, Kirikkale University, Kirikkale 71450, Turkey; 2 Department of Biostatistics, and Medical Informatics, Faculty of Medicine, Inonu University, Malatya 44000, Turkey; 3 Physical Education and Sports Teaching, Faculty of Sport Science, Inonu University, Malatya 44000, Turkey; 4 Department of Physical Education and Sport, Faculty of Education, University of West Bohemia, 30100 Pilsen, Czech Republic

**Keywords:** biogroup, maturity, muscle mass, talent identification, power, hand grip strength

## Abstract

**Simple Summary:**

Muscle strength increases with age, and the period in which the increase in muscle mass is highest is the growth and development period in adolescents. In this context, the improvement of muscle power and muscle strength in adolescents can be achieved with the development of simple motor skills. Research on the relationship between biological maturation, muscle strength, and muscle power was limited in adolescents, and this research will make an important contribution to the literature. In this research, the relationship between biological maturation and muscle strength and power was investigated. In conclusion, biological maturation was found to be significantly associated with muscle strength and power in adolescents.

**Abstract:**

The purpose of this study was to investigate the relationship between adolescents’ biological maturation level and their muscle power, as well as their overall muscle strength. Overall, 691 adolescents (414 boys and 277 girls) aged 12.01–11.96 (measured for body mass, body height as well as vertical jump, muscle power, and muscle strength). There was a statistically significant difference in terms of average right and left grip strength, vertical jump, and power in the late maturation group. For the body height and vertical jump averages in male adolescents, it was observed that the body height and vertical jump averages in the late group were significantly lower than in the early and on-time maturation groups. For female adolescents’ chronological age, sitting height, body mass, BMI, left and right grip strength, and power averages were found to be significantly higher compared with the on-time group (*p* < 0.05). It was established that biological maturation has a substantial link with vertical jump height and power, as well as grip strength on the right and left hands.

## 1. Introduction

Biological maturation, expressed as a process that characterizes human growth and development, is affected by individual differences and aims to progress toward the level of maturity [1,2]. The growth rate of children and the development of the organism are variable and it takes about 20 years for a newborn to complete the morphological, physiological, and psychological development process and reach biological maturity [1,3,4]. In this context, depending on biological maturation, the ability of a muscle to gain strength and power and develop rapidly increases until the age of 20 [5]. For this reason, determining the biological maturation level is important in terms of observing growth and performance development in order to objectively evaluate the competencies of talented young athletes [6,7]. The increase in muscle power and muscle strength in children is related to age, gender, growth level, and morphological characteristics [7].

Regular strength training improves adolescents’ muscle function [8]. Muscle strength increases with age, and the period in which the increase in muscle mass is highest is the growth and development period in adolescents [9]. In this context, the improvement of muscle strength and muscle power in adolescents can be achieved with the development of simple motor skills [10]. While the rate of increase in muscle strength and muscle power in girls and boys in preschool and primary school periods is similar, differences emerge with the onset of puberty [11]. Because girls reach puberty earlier than boys, they surpass boys in muscle strength and muscle power [12]. When we look at the following periods, as boys reach puberty, they increase in muscle strength and muscle power and exceed the level of girls [13]. Changes observed in terms of muscle power and muscle strength in children are significantly affected by factors such as growth and biological maturation [14]. Studies show that children who mature earlier than their peers are more developed (taller and heavier) in terms of both height and body weight than children who mature on-time and later [15,16].

When young athletes come together for competition and training, a grouping is traditionally based on chronological age (the age at which the individual was born) in order to provide a fair environment in terms of competition [3,6]. However, it should be noted that some characteristics of children of the same chronological age, such as muscle strength and power, may differ from each other, that some may or may not mature earlier than those in the same age group, and that there may be different physical and mental advantages or disadvantages among adolescents of the same age [4,6,9]. Therefore, reaching the best level of muscle power and muscle strength in adolescents has an important place in terms of development [17,18]. In many studies on athletic adolescents, it has been determined that those who mature early are stronger and taller than those who mature late and on-time, but when the literature is examined, there are limited studies on non-athletes. When the literature was examined, the biological maturity levels of adolescents also differ according to race [1,19]. This research will make a significant contribution to the literature since there was not much research on the relationship between biological maturation, muscle strength, and muscle power in Turkish adolescents. While the research focuses on athletic adolescents, there are very few studies on those who do not participate in sports. In this context, the aim of the study was to examine the relationship between biological maturation level and muscle strength and muscle power in adolescents.

## 2. Materials and Methods

### 2.1. Participants

A total of 691 adolescent participants were in the study [boys: n = 414, age = 12.02 ± 0.30 years; girls: n = 277, age = 11.96 ± 0.25 years]. This study was conducted in Turkey. All participants included in this study regularly engage in a physical education class for 2 h a week. Each participant in the study visited the laboratory before the tests. During the laboratory visit, the participants were informed about the research. At the next laboratory visit, the tests were carried out by the experts. Then, the biological maturation status of the participants was calculated. This study was conducted at Kirikkale University, Sports Sciences Faculty, according to the principles outlined by the Declaration of Helsinki. The ethics committee approved by the Kirikale University Non-invasive Research Ethics Committee (Date: 12 January 2022, Number: 2022-01-04). Parents were fully informed about the procedures of this study and signed written informed consent. All children and their parents were briefed on the measurement protocol and the purpose of the study. None of the participants in the measurements were excluded from the study. The G*power program was used to determine the appropriate sample size for the study. While the amount of Type I error (alpha) is 0.05, the power of the test (1-beta) is 0.80, and the effect size is 0.15, at least 432 participants should be included in the study according to the theoretical power analysis process applied using the one-way ANOVA [20].

### 2.2. Procedures

The testing sessions were performed in one day for every group at the university’s Exercise Physiology Laboratory and gymnasium. The first session of tests included measurements of anthropometrics before breakfast. Grip strength and countermovement jump (CMJ) tests were performed after adolescents were made to do 5 min of jogging at a low tempo, 2 min of free stretching, and 8 min of upper and lower extremity movements. The total warm-up time was set to 15 min with rest periods in a designated area of 10 m. The rest period of the participants was set to three minutes after each test [21]. Participants were instructed to follow guidelines before all tests: (a) participants were asked to wear shorts and T-shirts, (b) avoid vigorous exercise 24 h before laboratory tests, (c) Participants did not consumption coffee and tea before laboratory tests. All participants’ standardized procedures were followed for each assessment test, and they were asked to perform the following tests and measurements with maximum effort: body height, sitting height, body mass, right and left grip strength, and vertical jump. Anthropometric measurements and then physical performance tests were collected by the same trained team. Performance assessments (grip strength and power) were performed in an indoor gym with a three-minute rest period between each measurement.

### 2.3. Measurements

#### 2.3.1. Anthropometric Measurements

Participants’ height and sitting height were measured with a portable measure that can measure 0.1 cm (Seca 213, Hamburg, Germany). A Tanita Body Composition Analyzer BC 418 Professional model Japan was used to assess the body weight of the participants. Participants’ maturity status was estimated using the percent estimated adult height at observation (%PAS) [22]. The maturity level of each participant was categorized according to their %PAS z-score. Next, participants’ maturation was categorized as early (z-score > 0.5), on-time (z-score ± 0.5), and late (z-score < 0.5).

#### 2.3.2. Sitting Height Measurement

Participants were asked to sit straight on a chair. Thanks to the adjustable legs of the chair, it was adjusted according to the leg length of the participants. They were asked to take a deep breath and the value obtained was recorded in centimeters. The measurements were made with a balanced and easily moving height measuring instrument (Holtain brand stadiometer with 0.1 mm precision).

#### 2.3.3. Somatic Maturation

Predicted adult stature (PAS) was used as an indicator of maturation [22]. The child’s current height was then used, expressed as a percentage (%PAS) of the estimated adult height. The calculation is made by the PAS protocol, participants’ age (decimal), height, and average parental height. Information about the height of the parents was collected in the informed consent form. The PAS variable was expressed as a percentage of estimated adult height (APAS) [1]. Among adolescents of the same chronological age, individuals with higher estimated adult height are considered to be more advanced in physical maturation than lower individuals [22]. The Khamis–Roche method has been used in several studies to estimate biological maturity status [23,24]. In this study, the grouping was performed among children. Using the sample median z-score of the obtained %PAS, the latest ripening (*p* < 50%) and the earliest ripening (*p* > 50%) are given.

#### 2.3.4. Grip Strength

The participants’ maximal isometric grip strength was measured using a digital hand dynamometer (TKK-5401 Grip-D, Takei, Japan). After adjusting the hand dynamometer to the participant’s hand size, measurements were taken with the shoulders in 90° flexion and the elbow fully extended [25,26]. For standardization purposes, all participants were asked to start with their dominant hand for grip strength. Participants were asked to squeeze the handle of the handgrip dynamometer as hard as they could and maintain this effort for 5 s. During the test, the children were provided verbally motivated (i.g., squeeze as hard as you could). It was measured three times, alternating with 1-min intervals between trials, and the best grade was recorded [27,28].

#### 2.3.5. Countermovement Jump

The countermovement jump (CMJ) was conducted for monitoring, performance status in individual. A jumping mat was used to assess vertical jump (Smart Jump, Fusion Sport, Australia). Participants were asked to start with both feet on the platform in an upright position, then make a rapid downward movement towards a 90° knee angle, and jump as high as possible, and wait motionlessly on the platform until the computer beeps [29,30]. The participants made three jumps and the best value was used.

#### 2.3.6. Muscle Peak Power

Vertical jump and body weight values were used to calculate peak muscle strength. Peak power measurement was calculated according to the previously determined formula [31], [peak power = −1714.116 + [(47.788 ∗ body weight (kg)] + [(58.976 ∗ countermovement jump height (cm)]. 

### 2.4. Statistical Analysis

The conformity of the quantitative data to the normal distribution was evaluated with the Kolmogorov–Smirnov test [32]. Since the quantitative data showed a normal distribution (*p* < 0.05), they were summarized with mean and standard deviation. Independent-group t-test and one-way ANOVA test were used where appropriate for intergroup comparisons of data. Post-hoc analyses after the ANOVA test were performed with the Tukey test. The effect size (Cohen’s d) between the groups was interpreted as a small effect between 0.20–0.50, a medium impact between 0.50–0.80, and a large impact above 0.80 [33]. Pearson correlation coefficients were calculated to determine the direction and strength of the relationship between the variables. The *p* < 0.05 value was considered statistically significant in the analyses. The American Psychological Association (APA) 6.0 style was used to report statistical differences [34]. All analyzes were performed using Python 3.9 and IBM SPSS Statistics 28.0 for Windows (New York, NY, USA) software.

## 3. Results

Descriptive statistics of the chronological, anthropometric, and physical fitness tests by sex groups are illustrated in Table 1.

Table 2 shows the change in demographic information and fitness data of participants by sex. According to the results of the study, the mean age of the boys participating in the study was significantly higher than the girls in terms of sitting height, vertical jump, and power (*p* < 0.05).

Table 3 shows the change in demographic information and fitness data of participants according to maturity groups. According to the results of the study, among the maturity groups (early, on-time, and late), participants’ chronological age, body height, sitting height, body mass, BMI, grip strength right, grip strength left, vertical jump, and power averages were statistically significant (*p* < 0.05). Post-hoc analyses of chronological age, body height, sitting height, body mass, BMI, grip strength right, grip strength left, and power means showed that were significantly higher in the early group compared with the on-time and late groups. For vertical jump means, there was not a statistically significant difference between early and on-time groups (*p* > 0.05), but vertical jump means in the late group were significantly lower than in the early and on-time groups (*p* < 0.05). 

Table 4 shows the demographic information and fitness data of male participants according to maturity groups (early, on-time, and late). According to the results of the study, among the maturity groups, the male participants’ chronological age, body height, sitting height, body mass, BMI, grip strength right, grip strength left, vertical jump, and power results were statistically significant (*p* < 0.05). Post-hoc analysis: chronological age, sitting height, body mass, BMI, grip strength right, grip strength left, and power results in the early group showed that means were significantly higher compared with the on-time and late groups. There was no statistically significant difference between the early and on-time groups for the body height and vertical jump averages in boys participants (*p* > 0.05). However, body height and vertical jump results in the late group were significantly lower than in the early and on-time groups (*p* < 0.05).

Table 5 shows the demographic information and fitness data of female participants according to maturity groups. According to the results of the study, a statistically significant difference was found between the maturity groups for the chronological age, height, sitting height, body mass, BMI, grip strength right, grip strength left, vertical jump, and power results (*p* < 0.05). Chronological age, sitting height, body mass, BMI, grip strength right, grip strength left, and power results were significantly higher in the early maturation group than in the on-time maturation group.

**Figure 1:**
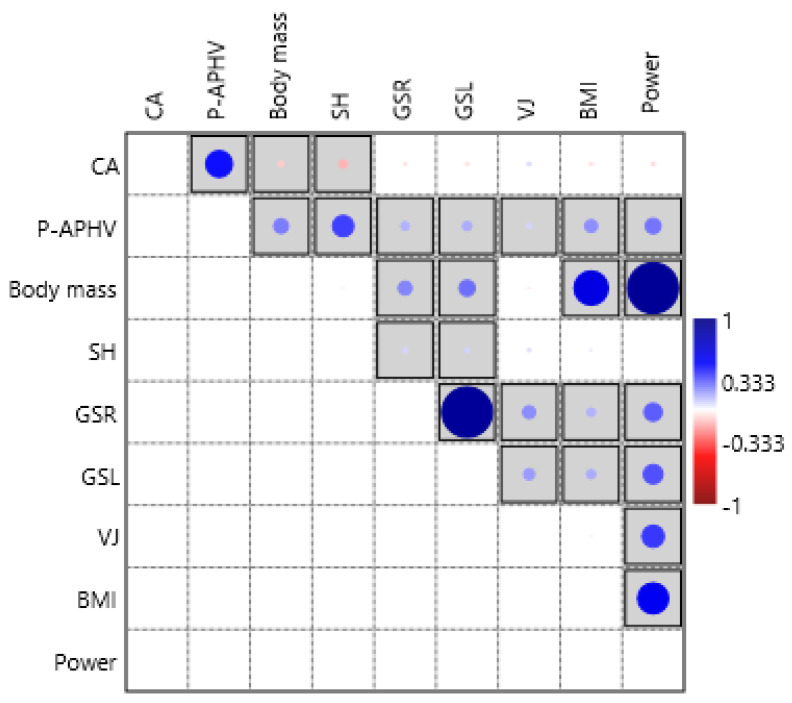
Correlation plot between power and other variables (correlations with significant *p* values are shown in the box).

When Figure 1 is examined, it is seen that there is a strong positive relationship between power, body mass, and BMI, and this relationship is statistically significant (*p* < 0.05). In addition, it can be said that the power will increase as the grip strength right, grip strength left, and vertical jump increase.

## 4. Discussion

The objective of this research is to examine the association between adolescents’ level of biological development and their overall muscle strength. According to the results of the research, the averages of the child’s chronological age, body height, sitting height, body mass, BMI, grip strength right, grip strength left, vertical jump, and power were statistically significant among the maturity groups (early, on-time, and late). Post-hoc analyses of chronological age, body height, sitting height, body mass, BMI, grip strength right, grip strength left, and power revealed that they were substantially greater in the early group than the on-time and late groups. Furthermore, there was a statistically significant difference in terms of average grip strength on the right, grip strength on the left, vertical jump, and power in the late group. For the body height and vertical jump averages in male participants, it was observed that the body height and vertical jump averages in the late maturity group were significantly lower than in the early and on-time groups. According to the results of the study, chronological age, body height, sitting height, body mass, and BMI of female participants among maturity groups (early, on-time and late), there were statistically significant differences found in grip strength right, grip strength left, vertical jump, and power. For female participants, chronological age, sitting height, body mass, BMI, grip strength right, grip strength left, and power averages were found to be significantly higher compared with the on-time group.

Albaladejo-Saura et al. (2022) [35] examined the impact of birth quartile, age, and biological maturation on the variations in kinanthropometric and physical fitness profiles between male and female adolescent volleyball players. The male players had higher values for the variables connected to bone and muscle, as well as the physical tests related to strength and power production. It was established that age, maturity offset, and birth quartile were all factors that had a statistical impact on the differences that were found between different sex groups. Age and biological maturity were shown to have a clear impact on the discrepancies that were found between the sexes in adolescent volleyball players. The researchers came to this conclusion after finding that there was a connection between the two factors [35]. In terms of maturity and performance parameters of individuals of the opposite sex, this study is comparable to the one we conducted. As a consequence of this, it was observed that performance parameters improved despite the fact that the participants in both studies were of different sex. Almeida-Neto et al. (2021) [36] examined the predictive power of the biological maturation (BM) markers (peak height velocity (PHV) and bone age (BA)) and lean body mass (LM) in connection to upper and lower limb muscular power and upper limb muscle strength in teenage athletes at puberty. They reported that LM, BA, and PHV were related to HG and ULS in both sexes. In both sexes, BA was related to vertical jump (VJ) and countermovement jump (CMJ). In both sexes, LM was found to be associated with BA and PHV. Analysis using a multilayer artificial neural network (MLP) showed that the LM provides a probability of more than 72% to predict the muscle power of upper and lower limbs, as well as the strength of the upper limbs; on the other hand, the PHV provides a probability of more than 43%, and the bone age provides a probability of more than 64% in both female and male adolescent athletes [36]. Although this study does look at several performance parameters in terms of maturity, the only way in which it is comparable is in terms of the contribution it makes to the growth of vertical jump performance. This does not qualify as a study that includes participants of different sexes. Massa et al. (2022) [37] examined the effect that birth date, salivary testosterone [sT] concentration, sexual maturity status, and overall strength had on the selection phase of an elite Brazilian soccer team over a period of twelve months. This was the second part of a selection phase that lasted for twenty-four months. They show that birth date and biological maturity have a significant impact on the selection process for young soccer players [37]. Taking into account the findings of this study, it appears that biological maturity has a significant impact on the selection of young soccer players. In light of these findings, soccer coaches should be aware of the impact of these characteristics in order to more effectively pick players. A similar point of view is presented as a result of our study. Guimares et al. (2019) [38] examined the effects of age, maturity status, anthropometrics, and years of training on the physical performance and technical skill development of male basketball players ranging in age from 11 to 14 years old. It determined how much maturity level and number of years spent training contributed to players’ overall levels of physical and technical performance. According to the findings, persons who reached their full maturity at an earlier age were larger in size, weighed more, and possessed greater levels of strength, power, speed, and agility. Early developing people continued to exhibit superior levels of power, swiftness, and agility even when age, height, and body mass were considered. In addition to that, they had a greater performance in the test of rapid-fire shooting. Aside from assessments of aerobic fitness, abdominal muscular strength and endurance, and lower body explosive power, the most important factor that contributed to the variety in physical performance tests was the participants’ levels of maturity [38]. The similarities between this study and ours are that the early maturing individuals in our study are taller, heavier, and have stronger grip strength in both hands than the individuals in the findings. De Almeida-Neto (2022) [39] investigated the impact of bone mass on upper and lower limb muscle strength in male and female adolescent athletes and non-athletes. The upper limb strength had a significant effect on the bone mass of adolescent athletes of both sexes. According to the study’s findings, the muscular strength variables had a large effect size on the bone mineral density (BMD) and bone mineral content (BMC) of male and female athletes, regardless of the sports group. Additionally, it was found that maturation has a large effect size on the bone mass of female athletes, but chronological age has a large effect size on the bone mass of male athletes. In contrast, for the control group of both sexes, chronological age, maturity, and characteristics associated with muscle strength had large effects on BMD and BMC [39]. A phenomenon similar to our study is the effect of biological maturity on vertical jump and hand grip strength. Gómez-Campos et al. (2018) [40] examined the hand grip strength (HGS) of students based on their chronological and biological ages to develop normative criteria for children and adolescents in Chile. From 13 to 17 years of age, boys demonstrated more HGS than girls. There were also significant differences between the sexes and at all levels of biological age. In conclusion, HGS during childhood and adolescence should be analyzed and interpreted based on biological age rather than chronological age [40]. In addition, it can be said that this study is similar to our study in terms of the conclusion that biological maturation has an important relationship with right grip strength and left grip strength. Jones et al. (2000) [41] conducted an investigation to see how performance in physical fitness tests was affected by sexual maturity. They reported that variations in female participants’ physical performance throughout maturation are mostly caused by changes in mass and body height, but there are some qualitative differences in performance related to other factors in boys [41]. When examined in terms of the common point with our study, it can be reported that it is similar to our study due to the effects of biological maturity on physical fitness test performance (hand grip strength and vertical jump test results).

The results of this study are subject to several limitations. In this research, biological maturation was also achieved based on predictive models (i.g., hand and wrist X-rays and longitudinal monitoring of the onset of puberty in those evaluated). Also, in this study, PP was evaluated using the estimation formula. Better results could have been obtained if the force platform, which is a gold standard measurement method, was used. Another limitation of our study was that the measurement of hand grip strength was started with the dominant hand, different results could be obtained if it was started with a random hand.

## 5. Conclusions

The main finding of this study was that biological maturation was associated with PP and right- and left-hand grip strength. In addition, the CMJ values of male participants who matured early and on-time were found to be better than those of late biological maturation adolescents. Considering the research results, we can say that early biological maturation in adolescents is more advantageous. If late-maturing adolescents do not engage in regular physical activity, muscle functions can be improved by directing them to perform physical activity. Future research may examine the underlying mechanisms of the late biological maturation of adolescents.

## Figures and Tables

**Table 1:** Descriptive statistics (minimum, maximum, mean, and standard deviation) of the chronological variables, anthropometric profile, and physical fitness tests by sex groups.

Variables	Sex									
	Girls (n = 277)					Boys (n = 414)				
	Mean	SD	95% CI	Min	Max	Mean	SD	95% CI	Min	Max
Chronological age (years)	11.96	0.25	[11.93–11.99]	11.52	12.68	12.02	0.30	[11.98–12.05]	11.51	12.50
Percentage of PAS (%)	85.04	6.58	[77.29–92.78]	69.50	98.70	84.85	2.71	[84.53–85.17]	75.40	92.60
Body height (cm)	155.66	7.94	[154.72–156.59]	130	175	155.23	9.10	[154.16–156.30]	115	175
Sitting height (cm)	76.19	5.01	[75.60–76.78]	66.90	88.20	77.35	5.00	[76.76–77.94]	66.90	88.20
Body mass (kg)	49.64	11.90	[48.23–51.04]	27.00	88.00	50.20	12.57	[48.71–51.68]	27.50	92.00
BMI (kg/m^2^)	20.47	4.02	[19.99–20.94]	12.49	33.29	20.51	4.35	[19.99–21.02]	12.96	39.64
Grip strength right (kg)	22.22	5.37	[21.59–22.85]	10.20	40.30	22.39	5.46	[21.74–23.03]	7.90	55.80
Grip strength left (kg)	20.96	5.55	[20.31–21.61]	8.20	45.40	21.08	5.35	[20.45–21.71]	8.20	52.30
Vertical jump (cm)	21.54	4.43	[21.02–22.06]	10.20	34.10	22.74	4.66	[22.19–23.29]	8.30	34.70
Power (Watt/kg)	1928.51	630.50	[1854.26–2002.76]	422.55	3700.24	2025.99	640.95	[1950.51–2101.47]	468.41	4086.01

PAS (predicted adult Body height).

**Table 2:** Descriptive statistics of boys and girls and sex differences.

Variables	Girls (n = 277)	Boys (n = 414)	t-Value	*p*-Value	ES
Chronological age (years)	11.96 ± 0.3	12.01 ± 0.3	−2.467	0.014 *	0.167
**Anthropometry**					
Body height (cm)	155.6 ± 7.9	155.2 ± 9.1	0.654	0.520	0.046
Sitting height (cm)	76.2 ± 5.0	77.3 ± 4.9	−2.986	0.003 *	0.223
Body mass (kg)	49.6 ± 11.8	50.2 ± 12.6	−0.587	0.553	0.049
BMI (kg/m^2^)	20.5 ± 4.0	20.5 ± 4.3	−0.137	0.889	0
**Fitness**					
Grip strength right (kg)	22.2 ± 5.4	22.4 ± 5.5	−0.401	0.688	0.037
Grip strength left (kg)	21.0 ± 5.6	21.1 ± 5.4	−0.295	0.768	0.018
Vertical jump (cm)	21.5 ± 4.4	22.7 ± 4.7	−3.376	0.001 *	0.262
Power (Watt/kg)	1928 ± 630	2025 ± 640	−1.972	0.048 *	0.153

BMI; Body mass index, ES; Cohen’s d effect size, *; statistically significant (*p* < 0.05).

**Table 3:** Descriptive statistics of boys and girls by maturity status and the results of ANOVA test, post-hoc comparisons, and effect size results.

Dependent Variables	Maturity Groups (n = 691)			ANOVA			ES		
	Early (n = 114)	On-Time (n = 538)	Late (n = 39)	F	*p*-Value	Post-Hoc Comparisons	Early On-Time	Early Late	On-Time Late
Chronological age (years)	12.1 ± 0.3	11.9 ± 0.3	11.9 ± 0.3	9.441	0.001 *	Early > on-time & late	0.31	0.57	0.50
**Anthropometry**									
Body height (cm)	160.3 ± 8.6	154.8 ± 8.4	149.5 ± 8.1	31.276	0.001 *	Early > on-time > late	0.57	1.03	0.92
Sitting height (cm)	81.6 ± 3.9	75.9 ± 4.7	76.1 ± 4.2	71.266	0.001 *	Early > on-time & late	0.87	1.56	1.39
Body mass (kg)	59.7 ± 12.1	48.1 ± 11.3	47.8 ± 12.6	47.970	0.001 *	Early > on-time & late	0.71	1.28	1.14
BMI (kg/m^2^)	22.9 ± 4.4	20.1 ± 4.0	19.0 ± 4.1	24.485	0.001 *	Early > on-time & late	0.51	0.91	0.82
**Fitness**									
Grip strength right (kg)	24.8 ± 3.9	21.9 ± 5.7	20.9 ± 3.7	14.915	0.001 *	Early > on-time & late	0.39	0.71	0.64
Grip strength left (kg)	23.5 ± 3.9	20.6 ± 5.7	19.5 ± 3.5	15.790	0.001 *	Early > on-time & late	0.40	0.73	0.65
Vertical jump (cm)	23.2 ± 4.5	22.2 ± 4.7	19.9 ± 3.0	7.819	0.001 *	Early & on-time > late	0.28	0.51	0.46
Power (Watt/kg)	2506 ± 621	1894 ± 585	1742 ± 636	53.239	0.001 *	Early > on-time & late	0.75	1.35	1.21

APHV: age at peak height velocity, BMI; Body mass index, ES; Cohen’s d effect size, *; statistically significant (*p* < 0.05).

**Table 4:** Descriptive statistics of boys by maturity status and the results of ANOVA test, post-hoc comparisons, and effect size results.

Dependent Variables	Maturity Groups (n = 414)			ANOVA			ES		
	Early (n = 53)	On-Time (n = 322)	Late (n = 39)	F	*p*-Value	Post-Hoc Comparisons	Early On-Time	Early Late	On-Time Late
Chronological age (years)	12.2 ± 0.3	12.0 ± 0.3	11.9 ± 0.3	10.371	0.001 *	Early > on-time & late	0.47	0.67	0.54
**Anthropometry**									
Body height (cm)	157.6 ± 10.9	155.5 ± 8.7	149.5 ± 8.1	10.076	0.001 *	Early & on-time > late	0.47	0.66	0.53
Sitting height (cm)	83.7 ± 2.5	76.5 ± 4.6	76.1 ± 4.2	65.185	0.001 *	Early > on-time & late	1.19	1.70	1.36
Body mass (kg)	56.3 ± 13.5	49.5 ± 12.1	47.8 ± 12.6	7.632	0.001 *	Early > on-time & late	0.40	0.58	0.46
BMI (kg/m^2^)	22.8 ± 4.8	20.3 ± 4.2	19.1 ± 4.1	10.279	0.001 *	Early > on-time & late	0.47	0.67	0.54
**Fitness**									
Grip strength right (kg)	25.4 ± 4.5	22.1 ± 5.6	20.9 ± 3.7	10.596	0.001 *	Early > on-time & late	0.48	0.68	0.55
Grip strength left (kg)	24.0 ± 4.6	20.8 ± 5.5	19.5 ± 3.5	10.744	0.001 *	Early > on-time & late	0.48	0.69	0.55
Vertical jump (cm)	23.6 ± 5.0	22.9 ± 4.7	19.9 ± 3.0	8.691	0.001 *	Early & on-time > late	0.49	0.62	0.49
Power (Watt/kg)	2363 ± 686	2004 ± 612	1742 ± 636	11.927	0.001 *	Early > on-time & late	0.51	0.72	0.58

APHV; age at peak height velocity, BMI; Body mass index, ES; Cohen’s d effect size, *; statistically significant (*p* < 0.05).

**Table 5:** Descriptive statistics of girls by maturity status and the results of t-test, comparisons, and effect size results.

	Maturity Groups (n = 277)		t-Value	*p*-Value	ES
Dependent Variables	Early (n = 61)	On-Time (n = 216)			
Chronological age (years)	12.0 ± 0.3	11.9 ± 0.2	2.128	0.034 *	0.44
**Anthropometry**					
Body height (cm)	162.7 ± 5.6	153.7 ± 7.4	8.796	0.001 *	1.27
Sitting height (cm)	79.7 ± 3.9	75.2 ± 4.9	6.635	0.001 *	0.95
Body mass (kg)	62.6 ± 9.9	45.9 ± 9.7	11.658	0.001 *	1.71
BMI (kg/m^2^)	22.9 ± 3.9	19.8 ± 3.8	5.605	0.001 *	0.81
**Fitness**					
Grip strength right (kg)	24.2 ± 3.2	21.7 ± 5.7	3.275	0.001 *	0.47
Grip strength left (kg)	23.8 ± 3.2	20.4 ± 5.9	3.446	0.001 *	0.62
Vertical jump (cm)	22.9 ± 4.1	21.1 ± 4.5	2.846	0.005 *	0.40
Power (Watt/kg)	2630 ± 534	1730 ± 501	10.669	0.001 *	1.77

APHV; age at peak height velocity, BMI; Body mass index, ES; Cohen’s d effect size, *; statistically significant (*p* < 0.05).

## Data Availability

Data are available for research purposes upon reasonable request to the corresponding author.
